# CIPPN: computational identification of protein pupylation sites by using neural network

**DOI:** 10.18632/oncotarget.22335

**Published:** 2017-11-06

**Authors:** Wenzheng Bao, Zhu-Hong You, De-Shuang Huang

**Affiliations:** ^1^ Institute of Machine Learning and Systems Biology, School of Electronics and Information Engineering, Tongji University, Shanghai, China; ^2^ Xinjiang Technical Institutes of Physics and Chemistry, Chinese Academy of Science, Urumqi 830011, China

**Keywords:** disease, post translational modification, classification

## Abstract

Recently, experiments revealed the pupylation to be a signal for the selective regulation of proteins in several serious human diseases. As one of the most significant post translational modification in the field of biology and disease, pupylation has the ability to playing the key role in the regulation various diseases’ biological processes. Meanwhile, effectively identification such type modification will be helpful for proteins to perform their biological functions and contribute to understanding the molecular mechanism, which is the foundation of drug design. The existing algorithms of identification such types of modified sites often have some defects, such as low accuracy and time-consuming. In this research, the pupylation sites’ identification model, CIPPN, demonstrates better performance than other existing approaches in this field. The proposed predictor achieves *Acc* value of 89.12 and *Mcc* value of 0.7949 in 10-fold cross-validation tests in the Pupdb Database (http://cwtung.kmu.edu.tw/pupdb). Significantly, such algorithm not only investigates the sequential, structural and evolutionary hallmarks around pupylation sites but also compares the differences of pupylation from the environmental, conservative and functional characterization of substrates. Therefore, the proposed feature description approach and algorithm results prove to be useful for further experimental investigation of such modification’s identification.

## INTRODUCTION

Post-translational modifications results in various human diseases such as cancers and autoimmune diseases, pernicious anemia, cardiovascular disease, cancer and neurodegenerative disorders. Protein plays the key roles in the field of biology and disease. Such modifications provide a fine-tuned control of protein functions in various types of cells in the field of disease research and drug design. For example, the well-known tumor suppressor p53 is subject to many post-translational modifications, which have ability to altering its localization, stability and other related functions, thus ultimately modulating its response to various forms of genotoxic stress [1–4]. Therefore, p53 drives both the activation and repression of a large number of promoters, which ultimately define its tumor sup-pressor abilities [5–10]. It could not be ignored that the above mentioned tumor suppressor is a critical transcription factor in the field of post translational modification [11].

When it comes to the post translational modification, it seems to be essential for regulating protein functions in all living cells and organisms [12–14]. It should be noted that ubiquitylation may seem to be one of the most common type of protein post-translational modification [15]. Such type plays significant roles in the regulation of DNA repair, transcription and other cellular processions. On the other hand, ubiquitylation is critical in the several types of Human diseases, such as lung cancer, breast cancer, Type 2 diabetes and other complex diseases which have been serious threats to human health [16–20].

Recently, pupylation, which is a common modification type in the protein post translational modification, has been treated as the first PTM in prokaryotes [21, 22]. Similar to ubiquitin, prokaryotic ubiquitin-like protein (Pup) seems to attach to specific lysine residues. As the initially found the PTM small protein modification in prokaryotes, prokaryotic ubiquitin-like protein (Pup) in Mycobacterium tuberculosis (Mtb) play an important role in the selection of proteins’ degradation [23].

To better understand the biological mechanisms of pupylation, the basic target and fundamental task are the accurate and effective prediction of the pupylation sites. Another is worth mentioning, cellular pathways involved in determining the fate of essential proteins by PTM processions and events. Such pathways seem to be an increasingly important area of related study in the field. Among so many modifications, the better understanding of eukaryotic ubiquitylation by ubiquitin protein has shown to be especially essential and valuable [24–28]. With those capabilities and functionalities, such pathways play particular key roles in the cellular events [29–31].

Recently, several large-scale proteomics advanced technologies have been brought in identification pupylation sites [32–36]. Considering conventional experimental approaches’ weakness is usually costly and luxury. Therefore, it is urgent to design and develop computational methods to identify the potential pupylation sites. Up to now, several predictors have been proposed and developed for such events. When it comes to the group-based prediction system 2.2 versions (GPS2.2) algorithm, Liu and their coworkers introduced the first predictor for the prediction of the pupylation sites in the field of bioinformatics [37]. Yan Xu and their team developed the iSulf-Cys algorithm to identify the S-sulfenylation Sites with the physicochemical properties of amino acid residues [38, 39]. Tung developed a predictor, which is named the iPUP server, utilizing the composition of k-spaced amino acid pairs that are a special composition of amino acid and its abbreviation is CKSAAPs surrounding lysine-centered peptides with the SVM algorithm [40]. Chen and colleagues have designed a predictor on support vector machine named PupPred server, where the amino acid pair composition employed as the features so as to encode lysine-centered peptides [41]. Currently, Hasan and coworkers proposed a web server, which is named pbPUP, to predict pupylation modification sites with the method on profile-based CKSAAPs’ feature [42, 43]. And such model is also employed the SVM model as the classifier.

## RESULTS

By fusing three different and distinguish amino acid residues’ component information approaches, a new ensemble classification framework named, has been established for predicting pupylation sites in protein sequences. To evaluate the performance of the proposed two features, several parameters, including Sn, Sp, Acc, MCC and AUC have been employed as the in this work. The following equations, which include from eq.(1) to eq.(4), have the ability to demonstrate the function of the above mentioned parameters. All experiments are performed on the personal computer with a 3.40GHz Intel(R) Core(TM) i7-3770M CPU and 16G bytes of memory.

$$\mathrm{Sn}=\frac{TP}{TP+FN}$$ (1)

$$Sp=\frac{TN}{TN+\mathrm{FN}}$$ (2)

$$Acc=\frac{TP+TN}{TP+FP+TN+FN}$$ (3)

$$Mcc=\frac{TP*TN-\mathrm{FP}*\mathrm{FN}}{\sqrt{\left(TP+TN\right)*\left(TP+FN\right)*\left(TN+FP\right)*\left(TN+FN\right)}}$$ (4)

Where, the TP means the true sample in positive set, the TN means the true sample in the negative one, the FP means the false sample in the positive and the FN means the false sample in the negaitve. Meanwhile the AUC means the area under the ROC curse, which have the ability to show the receiver operating characteristic in the field of classification issue.

### Performance of AAIndex PCA

In our study, each type of features has contributed to the prediction model in different degrees. So, the employed feature types’ comparison showed in the Table 1. From the table, it was easily to find that the features on the amino acid upstream/downstream residues composition information play less significant effect in the pupylation sites prediction. In other words, the adjacent amino acid residues’ statistic features do not meet the needs on accurate and precise prediction pupylation sites. The second type of classification feature is the features derived from the AAIndex. These features contain the physical, chemical and biological properties of each kind amino acid residues. From the table, we can find that the candidate properties work well in this kind of post translational modification. However, the large amount of pupylation segments will cause the huge number of feature information. Such situation will also bring the unprecedented challenges in the field of computation, storing and transmission. The next type of feature is the AAIndex features’ combination by PCA. Such type of features seem to have the similar performances with the second features’ type. It was noted that the scale of these features is far smaller than the former one. Therefore, the AAIndex features’ combination with PCA has the ability to replacement the AAIndex features’ combination in some degree. Meanwhile, the PCA procession merely survives the main information of former combination. Some minor information of the AAIndex features’ combination will be taken into account in the future research.

**Table 1:** Prediction the database on Pupdb 10-fold with AAIndex PCA

Subset	Sn(%)	Sp(%)	Acc(%)	Mcc	AUC
1	65.21	96.45	80.83	0.6491	0.8017
2	73.42	95.36	84.39	0.7049	0.8115
3	69.43	97.56	83.50	0.6980	0.8231
4	64.43	96.23	80.33	0.6398	0.7667
5	72.02	98.32	85.17	0.7291	0.8091
6	65.32	97.67	81.50	0.6656	0.8073
7	68.64	97.53	83.09	0.6912	0.8137
8	69.43	98.64	84.04	0.7117	0.8342
9	67.57	98.67	83.12	0.6970	0.8451
10	77.71	98.63	88.17	0.7806	0.8072
Average	69.55	97.51	83.53	0.6987	0.8119

The first column records sensitivity of these ten subsets of the Pupdb. The second column records the specialty of such subsets. And the 3th and 4th column record the accuracy and the Markovian correlation coefficient, AUC of these data, respectively.

**Figure 1:**
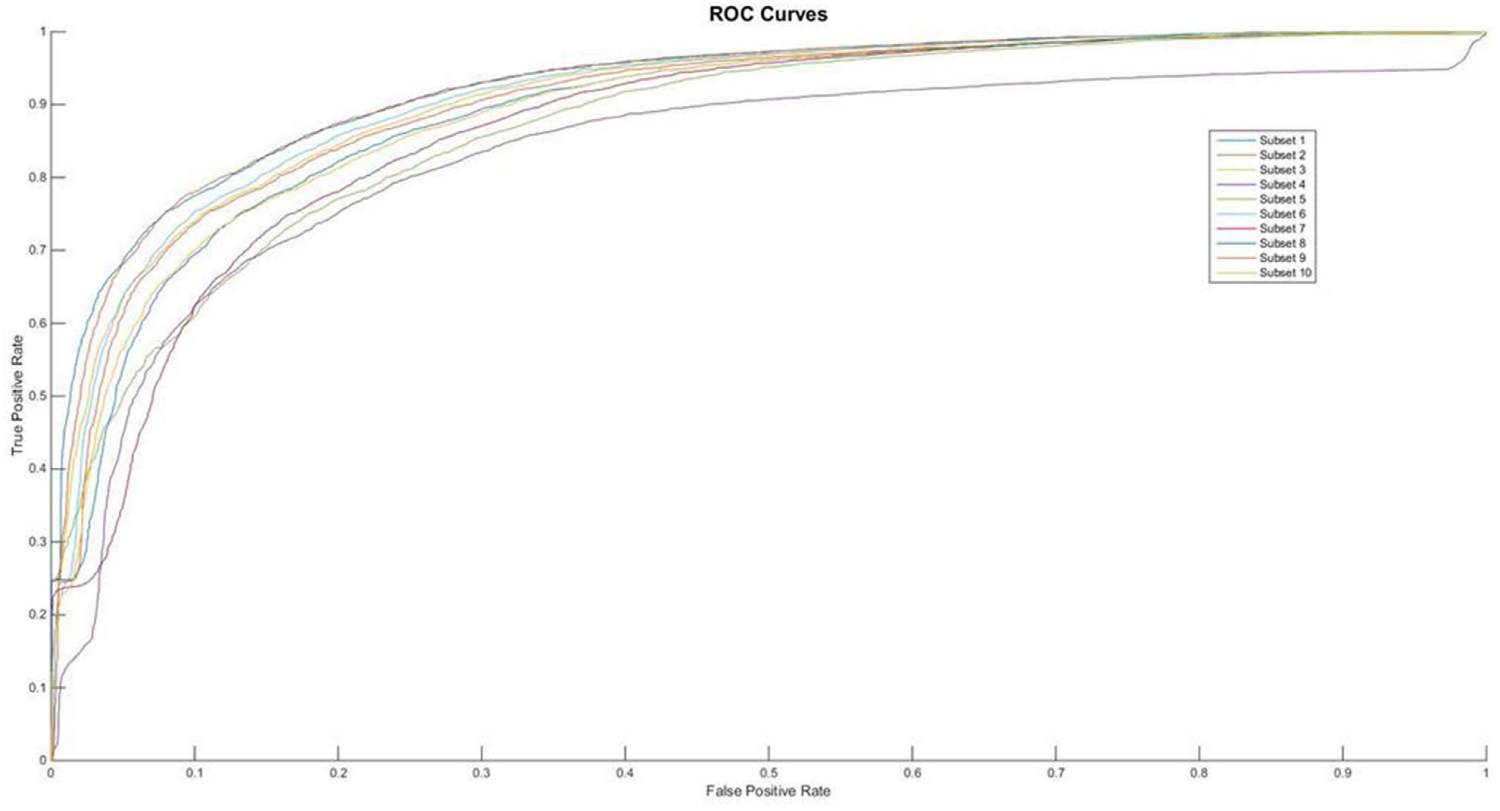
The ROC curves of feature of AAIndex PCA

In the aspect of neural network, we selected the optimal number of hidden neurons by testing from 2 to 5 with the alternative layers ranging from 2 to 4. The results of 10-fold validation were shown in Figure 1. The other performances’ measures are listed in Table 1.

### Comparison with other methods

To demonstrate the performance of proposed model, we compare current prediction model with the other models. Meanwhile, we also carry the comparisons among *k* nearest neighbors, support vector machine and Naïve Bayes classification algorithms in this work. The testing set was submitted to the GPS-PUP web server and the outputs were utilized to calculate the corresponding sensitivity, specificity and other performance indicators. It should be pointed out that we can guarantee that the testing data’s protein segments are not included in the training dataset of GPS-PUP.

During this work, it is found that the ensemble model affected by the random initializations similar to other machine learning algorithms. And then, we have repeated the experiments for several times with different initializations to demonstrate the stability of the proposed ensemble algorithm.

**Figure 2:**
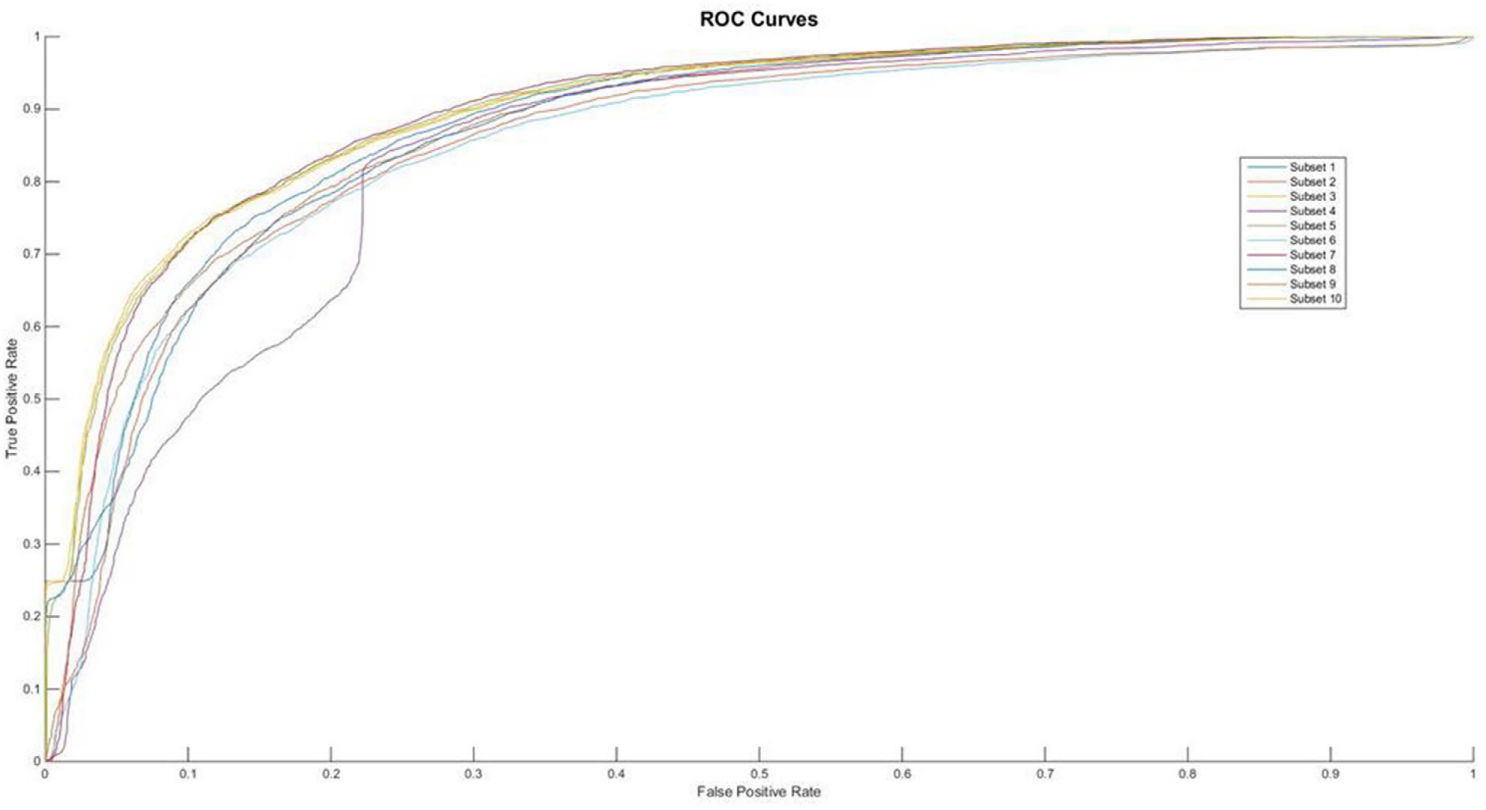
The ROC curves of feature of AAIndex BLOSUM62 PCA

On the other hand, it is also interesting to find from the Figure 2 that the number of hidden layers in the neural network of the proposed ensemble algorithm plays a critical role in its performance. Although this paper has tested a large range from 2 to 4 and selected 15 to construct our final classification model. Meanwhile, the selection of these parameters can also be applied independent. Hence, one of the important future research topics is to discover the size of hidden layers and hidden nodes with difference type data structures.

From the Table 1, we can find that the performances of feature AAIndex PCA can clear distinct the difference between the negative samples and the positive ones. It was pointed that the first proposed feature extracting method achieves the average Acc value of 83.41 in the PupDB data set, which can be treated as the benchmark data set in the field of identification pupylation sites. And the other performances on evaluating the method are Sn, Sp and Mcc, whose values are 69.55, 97.51 and 0.6987, respectively. So, in this 10-fold cross validation, the domain of Acc can range from 80.33% to 85.17. Meanwhile the Sn’s domain can range from 65.21% to 77.71%. And the upper bound and the lower bound of Sp are 98.67% and 95.36%, respectively. At the same time, it is easy to find out that the values of Sn are significantly higher than the Sp’s values in each subset. And the ROC curves of each subset show in the Figure 2.

### Performance of AAIndex BLOSUM62 PCA

From the Table 2, we can find that the performances of feature AAIndex BLOSUM62 PCA can clear distinct the differences between the negative samples and the positive ones. It was pointed that the first proposed feature extracting method achieves the average Acc value of 89.12 in the PupDB data set. And the other performances on evaluating the method are Sn, Sp and Mcc, whose values are 97.96, 80.28 and 0.7949, respectively. So, in this 10-fold cross validation, the domain of Acc can range from 83.82% to 92.59%. The range of Acc is much smaller than the AAIndex PCA method. Meanwhile the Sn’s domain can range from 87.57% to 99.81%. And the lower bound and the upper bound of Sp are 76.42% and 85.75%, respectively. However, it is interesting to observe that the values of Sp are significantly higher than the Sn’s values in each subset. And the ROC curves of each subset show in the Figure 2.

**Table 2:** Prediction the database on Pupdb 10-fold with AAIndex BLOSUM62 PCA

Subset	Sn(%)	Sp(%)	Acc(%)	Mcc	AUC
1	99.81	80.48	90.15	0.8183	0.8127
2	95.57	80.11	87.84	0.7660	0.8157
3	99.27	76.79	88.03	0.7806	0.8287
4	99.72	83.54	91.63	0.8437	0.7903
5	99.34	81.59	90.46	0.8224	0.8107
6	99.43	85.75	92.59	0.8599	0.8102
7	99.52	77.53	88.52	0.7898	0.8167
8	99.62	76.42	88.02	0.7817	0.8397
9	99.75	80.47	90.11	0.8175	0.8576
10	87.57	80.06	83.82	0.6782	0.8162
Average	97.96	80.28	89.12	0.7949	0.8199

The first column records sensitivity of these ten subsets of the Pupdb. The second column records the specialty of such subsets. And the 3th and 4th column record the accuracy and the Markovian correlation coefficient, AUC of these data, respectively.

In order to evaluate the performance of those two methods, several pupylation identification methods and algorithm have been developed in the website resources. However, some of them had broken links, so they could hardly be tested in this model. In fact the predictors, which employed PUL-PUP, PSoL, SVM_balance, Naïve Bayesian and other methods were included in the comparison tables. From the Table 3, we can find that the second proposed method can reach higher accuracy than the PUL_PUP method and the first method merely reach 82.51% in this performance. At the same time, we can also find that the methods such as the SET-SVM, IMP_PUP and the second method can get ideal values in the sensitivity and the methods such as the PUL-PUP, Naïve Bayesian and the first method can get appropriate value in the specialty.

**Table 3:** Prediction the Pupdb database comparison with other methods

Method	Sn(%)	Sp(%)	Acc(%)	Mcc	AUC
PUL-PUP	82.24	91.57	88.92	0.7413	0.7238
PSoL	67.50	73.60	70.55	0.4118	0.6378
SVM_balance	76.71	63.65	69.88	0.4071	0.6571
Naïve Bayesian	82.78	86.40	84.59	0.6923	0.7528
DEC–SVM	75.49	77.87	77.70	0.5338	0.7891
SET–SVM	93.77	77.87	79.05	0.7256	0.8013
IMP-PUP	94.58	78.12	79.34	0.7371	0.8031
AAIndex PCA+Neural Network	65.50	99.52	82.51	0.6914	0.8119
AAIndex BLOSUM62 PCA+ Neural Network	97.96	80.28	89.12	0.7949	0.8199

In order to evaluate the performance of those two features, several pupylation identification features also have been developed in the literature resources. In this work, several features such as Binary Encoding, AA Composition, AA Pair Composition, Grouping AA Composition, Physicochemical Properties, KNN Features, Secondary Tendency Structure and Binary Coding have been compared. The comparison among these features show in the Table 4.

**Table 4:** The comparison with difference features

Features	Sn(%)	Sp(%)	Acc(%)	Mcc	AUC
Binary Encoding	43.36	75.80	59.58	0.2026	0.6472
AA Composition	64.14	52.79	58.46	0.1704	0.6121
AA Pair Composition	62.46	62.48	62.47	0.2494	0.6917
Grouping AA Composition	41.78	76.04	58.91	0.1897	0.5919
Physicochemical Properties	55.53	63.93	59.73	0.1953	0.5976
KNN Features	64.94	55.85	60.39	0.2088	0.6477
Secondary Tendency Structure	59.96	57.40	58.68	0.1737	0.6211
PSSM	51.20	69.39	60.30	0.2094	0.6374
Binary Coding	64.04	78.60	71.63	0.4310	0.6271
PSSM2	61.11	68.94	65.11	0.3014	0.7921
AAIndex PCA	65.50	99.17	82.32	0.6868	0.8119
AAIndex BLOSUM62 PCA	97.96	80.28	89.12	0.7949	0.8199

## DISCUSSIONS

### Features

Generally, the types of proteins’ features can reach more than 10,000. Such huge of features, including statistical features such as amino acid compositions (AAC), dipeptide compositions (DC), biological features such as pseudo amino acid compositions (PseAAC), characteristic features such as hydrophilic, free energy of molecules and Van der Waals forces of amino acid residues and physical features such as relative molecular mass, molecular charge number and other relative features merely contain remarkably few key classification information in the prediction issue [68–70]. Nevertheless, the above mentioned features can hardly effectively and accurately have the ability to description the interaction between predicted modification lysine residue and upstream/downstream amino acid residues [71]. Therefore, a special type of features, utilized to classify and distinguish the pupylated lysine residues and the non-pupylated lysine residues, has been improved and polished in the proposed prediction method in this work.

Because of the potential sites, the features of amino acid residues should be taken into account. The most popular and well-known amino acids’ feature index is the AAIndex, which is a website database of numerical indices representing various physical, chemical and biological properties of the amino acid residues, pairs of amino acid peptides, other forms of protein sequence information. All those relative information could be easily derived from published literatures [72–74]. So, several types of amino acids’ features have been employed in this research. And the more detailed information on the selected amino acid features showed in Table 5.

**Table 5:** The selected properties from the AAIndex database

No.	AAIndex ID	Name of Properties
1	CHOP780207	Normalized frequency of C-terminal non helical region
2	DAYM780201	Relative mutability
3	EISD860102	Atom-based hydrophobic moment
4	FAUJ880108	Localized electrical effect
5	FAUJ880111	Positive charge
6	FINA910103	Helix termination parameter at position j-2, j-1, j
7	JANJ780101	Average accessible surface area
8	KARP850103	Flexibility parameter for two rigid neighbors
9	KLEP840101	Net charge
10	KRIW710101	Side chain interaction parameter
11	KRIW790102	Fraction of site occupied by water
12	NAKH920103	AA composition of EXT of single-spanning proteins
13	QIAN880101	Weights for alpha-helix at the window position of -6

**Figure 3:**
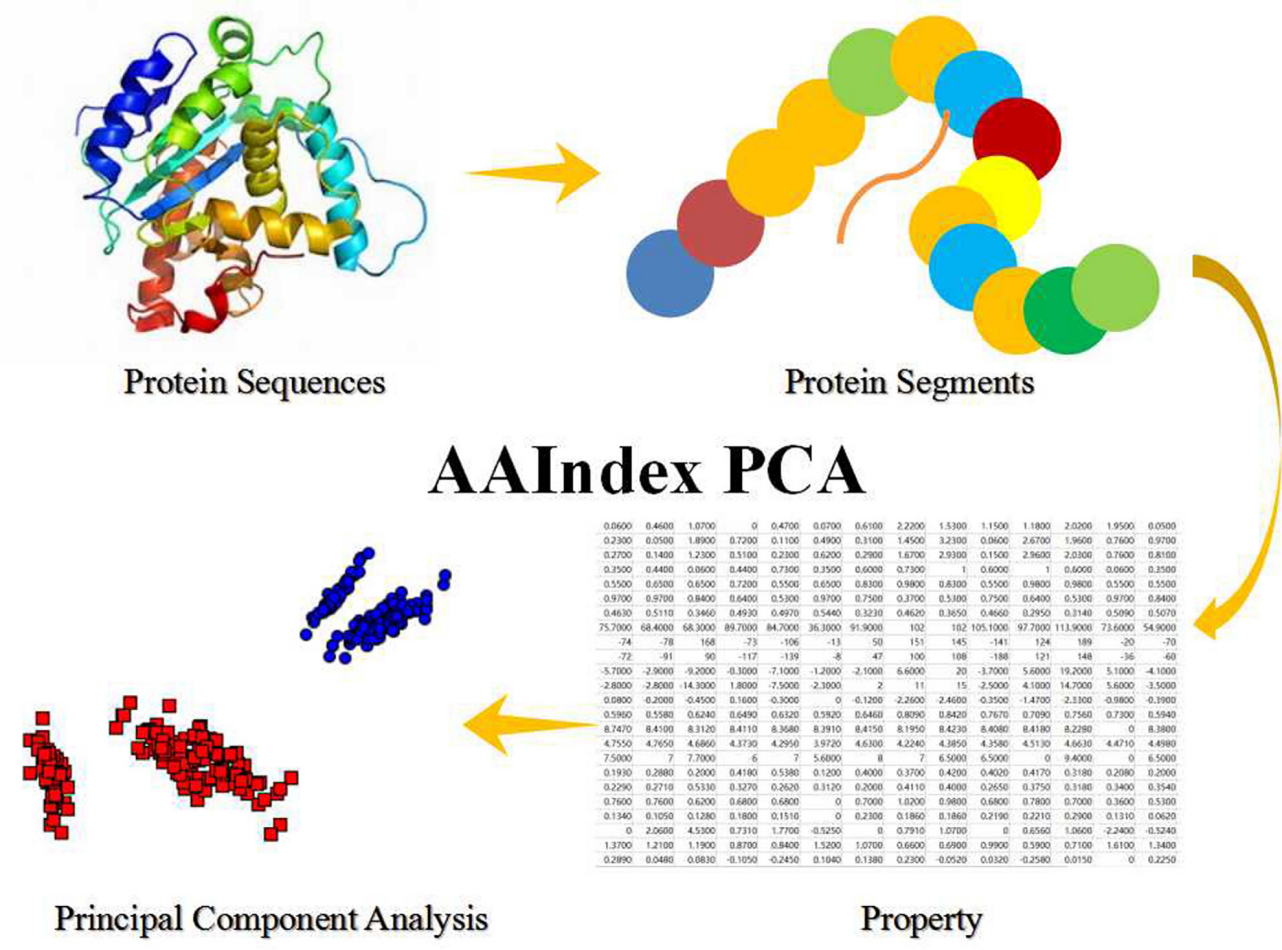
The Steps of AAIndex PCA Features The initial step is the predicted protein sequences in this work. The second step is the predicted amino acid segments from the protein sequences. The 3th step is transform the amino acid segments to property matrix of the amino acid segments. The fourth step is the Principal Component Analysis (PCA) of the property matrix.

In this work, we have selected several properties, which show in the Table 5, from the AAIndex database. Those selected properties have been constructed a matrix, whose size is m lines and *n* columns. The m lines mean the *m*-length predicted protein segment and the n-columns mean the n-dimension selecting property in this research. However, the property matrix seems to be hardly treated as the feature in this classification model. Therefore, the PCA (Principal Components Analysis) has been employed as the feature processing. PCA is a mathematical algorithm that tries to reduce and decrease the dimensionality of the data matrix. The detailed steps show in Figure 3.

Given a predicted sample matrix with m amino acid residues and n properties, the matrix is first focused on the means of variables. This will make sure the data have the ability to centering on the origin of principal components, and the data could not be affected by the spatial relationships of the data nor the variances along other variables [74–76]. The principal components *Y* is given by the linear combination of the variables *x*_1_, *x*_2_, …, *x*_m_ and the formulate shows in the (5).

$$Y=a_{1}x_{1}+a_{2}x_{2}+\ldots a_{m}x_{m}$$ (5)

The principal component is computed such that it accounts for the most possible variance of the selected properties. To prevent such state, weights are evaluated by the constraint that the sum of squares is equal to 1. And the formulate shows in the (6).

$$a_{1}^{2}+a_{2}^{2}+\ldots +a_{m}^{2}=1$$ (6)

**Figure 4:**
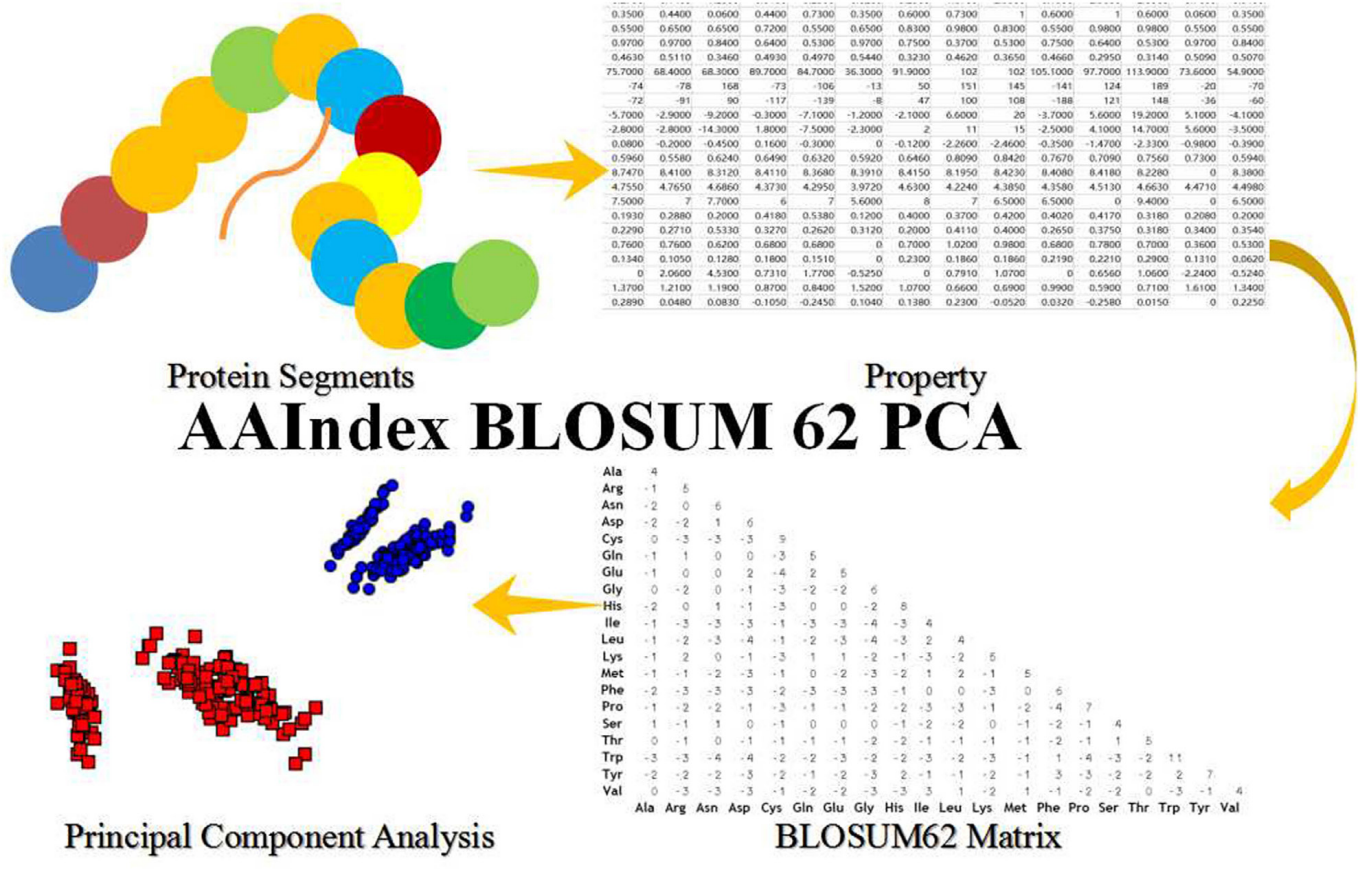
The Steps of AAIndex BLOSUM62 PCA Features The initial step is the protein segments of the predicted amino acid segments in this work. The 2nd step is transform the amino acid segments to property matrix of the amino acid segments. The first and second steps are same as the second and third steps of the steps of AAIndex PCA features. The 3th step is the BLOSUM 62 matrix, which is the interaction between the amino acid residues. The property matrix and the BLOSUM 62 matrix get the multiplication operation in this steps. And then, they get a novel interaction matrix. The fourth step is the Principal Component Analysis (PCA) with the novel interaction matrix.

In this paper, we took advantage of the BLOSUM62 matrix, a popular substitution matrix used for sequence alignment of proteins. This explains some details in BLOSUM62 that may seem counter intuitive at first glance. For example, W/W combination score +11 and L/L pair only score +4. The scores could be evaluated by the following equation. Those scores consist of a 20×20 score matrix. In our work, the values of BLOSUM62 are treated as the weights between the potential predicted lysine sites and the adjacent amino acid residues. The second type of feature is the first type feature with the relation weight between the lysine and other kinds of amino acid residues in the predicted protein segments. In order to show the steps more clearly, the following Figure 4 will be described the steps.

## MATERIALS AND METHODS

### Data

The post translational modification resources show the detailed system flow of the online-construction. Considering the inaccessibility of database, it contents in several online PTM resources, 11 biological databases related to PTMs are integrated in dbPTM totally and several biological processions [44–47].

First of all, a series of keywords, which is related to the PTM-related terms, have been constructed by referring to the UniProtKB and SwissProt resources on the PTM list [21, 48, 49]. At the same time, the detailed information of those databases has been annotated by the RESID that is another international protein database in the field of proteomics [50–54].

Next, all fields could be searched by a series of keyword list of the constructed table list in the PubMed and other proteomics databases. According to not complete count, to 2016, about 850 review and original articles associated with MS/MS proteomics and protein modifications are retrieved from those database. Therefore, those datasets of pupylated proteins and pupylation sites identified by large-scale proteomics experiments are extracted from various PTM databases [55]. Particularly, PupDB, which is a collection of pupylated proteins and pupylation sites, have been constructed by Tung and co-workers in 2012 [56, 57]. Such database includes 76, 51 and 55 pupylated proteins with known and reported pupylation sites in many datasets. Considering pupylation’s occurrence on lysine residues, both positive and negative sample groups with silicon methods are represented as 2*m*+1 length residues’ peptide segments with lysine in the center. The potential peptides with pupylated lysine in the center could be treated as positive samples. On the contrary, the other non-modified potential peptides seem to be the negative ones. At the same time, another step of the preprocessing seems to avoid overestimating prediction performances of proposed methods in this work. So the redundant peptides of identical sequences have been removed.

To solve the heterogeneity among those data collected from different databases from the website resource, such reported sites have been mapped from the UniProtKB protein. With the development of high-throughput of MS-based approaches in the field of post-translational proteomics, this update, meanwhile, includes manually curated MS/MS-identified peptides associated with PTMs from research articles [58–63].

The source of pupylation protein sequences have been extracted by the UniProtKB/Swissprot database in this research [64]. To ensure the quality, the selected data, have been used in this research, was constructed by the UniProtKB/SwissProt at http://www.ebi.ac.uk/uniprot/.

The detailed procedures show as following the steps:

- Vistiting the website at http://www.uniprot.org/, and then the button ‘Advanced’.
- Choosing the ‘Modified residue’ for ‘Fields’.
- Choosing the ‘Any experimental assertion’ for ‘Evidence’.
- The proteins thus obtained were subject to a screening operation to remove those sequences, which have above 50% pairwise sequence identity to any other.

It was pointed that the aforementioned existing prediction servers were generally trained about the experimentally annotated pupylated proteins. However, those prediction servers’ data resources have been collected from the PupDB database, which is a classical benchmark database [6]. It is noteworthy that only 268 annotated pupylated proteins with 311 known pupylation sites were included in the current version of PupDB database [65–67]. Considering such phenomenon, the scale of defined and submitted the modified protein sequences seem to be relatively small. Those prediction models and relative researcher could hardly reflect the real distribution of modification sites commendably. Consequently, the prediction accuracy of existing computational methods could hardly be unsatisfactory. Really, there are 268 annotated pupylated protein sequences.

In this study, the proposed method, which aims to improve the prediction of pupylation sites, by using an alternative structure neural network and employed two types of protein information as the classification features. Specifically, the alternation structure neural network classification model is trained on those training proteins segments taking advantage of the selected features. And the initial ensemble model is utilized to classification the testing pupylated proteins segments. Then, the final ensemble classification, which is used to construct the proposed algorithm, results at the end of classification. As illustrated by our experimental results, the performance of the predictor has been improved effectively by the selected data set. The results indicated that the proposed algorithm outperforms three other existing predictors significantly.

## CONCLUSIONS

Much knowledge about protein sequences with pupylation has been accumulated to date. There are still numerous unanswered issues and questions regarding specific aspects of the classification issue in the field of machine learning. Nowadays non-consensus sequences that make up their mind which specific lysine would become pupylation could be identified when non-homologous proteins seem to be considered. It is hard to regard that all segments carry similar structures before they bind to the component of the pupylation modification.

Systematic analysis of the pupylated sites along with information on the exact sites is utilized by identifying the modified sites from the protein sequences. Here, it can be easily find that not only the sequence markers but also structural markers about pupylated sites. First of all, the analysis of sequence features demonstrates that the adjacent amino acid residues in the potential segments could be close to modified lysines residues in spatial structure. Secondly, pupylation protein segments have high propensity flexibility in the field of protein structure. Finally, the conservative in pupylation segments seem to be high.

On the other hand, another significant result of this research is design of the pupylation sites prediction model with different types of features. Every selected type of features is contributed to the prediction model more or less. Here, it was pointed that unbalanced datasets, which the negative samples can reach 5 times than the positive ones, present a hottest topic in the field of machine learning classification. In our work, the unbalanced datasets will try to avoid the negative impacts with the preprocess steps, which the positive samples replicate themselves until the size of positive samples can generally reach the scale of the negative ones in both training and testing set. Nevertheless, the preprocess method will increase the burden of classification model. The model’s training time will be greatly extended. Considering the burden and the training time, an improved preprocess step has been introduced to deal with the unbalance classification model. Such improved step merely replicate the positive samples in the testing set. With such step, the unbalanced classification issues can be solved basically and the burden of classification model will not increase. For future research, other methods, such as semi-supervised learning, will be explored and developed to deal with the unlabeled post translational modification sites in the predicted protein segments.

To summarize, the design of ensemble classification model represents an attempt to predict candidate pupylated segments based on the multi-type feature. Because the size of experimentally identified modification sites will be rocketing in the future and such sites will be enriched the training set, the current accuracy of the ensemble is helpful to identify the new sites. With the established link between the feature description and the classification system, such predictions, especially when confirmed by experiments, would be helpful to identify the degradation possibilities of individual proteins more precisely, and may ultimately lead to design of drugs and treatment of diseases.

In this work, we have developed a novel pupylation sites prediction ensemble algorithm. To our knowledge, it is the first time such ensemble flexible neural tree model has been applied to predict the potential pupylation sites. Experimental results demonstrate that such method outperformed the existing pupylation sites prediction. At the same time, the majority modification type likely pupylation sites could be predicted in non-annotated lysine sites by utilizing the proposed ensemble model. Meanwhile, it could be believed that such method can be utilized to prediction the other types of modified sites in the potential protein segments. Therefore, we will design and develop the web server for such algorithm in future research.
